# *MTHFR* Gene Polymorphisms Prevalence and Cardiovascular Risk Factors Involved in Cardioembolic Stroke Type and Severity

**DOI:** 10.3390/brainsci10080476

**Published:** 2020-07-24

**Authors:** Dana Simona Chita, Anca Tudor, Ruxandra Christodorescu, Florina Nicoleta Buleu, Raluca Sosdean, Sanda Maria Deme, Simona Mercea, Adina Pop Moldovan, Ana Maria Pah, Any Docu Axelerad, Daniel Docu Axelerad, Simona Ruxanda Dragan

**Affiliations:** 1 Department of Neurology, Arad County Emergency Clinical Hospital, 310158 Arad, Romania; danaioncu@yahoo.com (D.S.C.); sandademe@yahoo.com (S.M.D.); 2 Department of Cardiology, “Victor Babes” University of Medicine and Pharmacy, 300041 Timisoara, Romania; buleu.florina@gmail.com (F.N.B.); ralusosdean@yahoo.com (R.S.); ana11p@yahoo.com (A.M.P.); simona.dragan@umft.ro (S.R.D.); 3 Department of Functional Sciences, “Victor Babes” University of Medicine and Pharmacy, 300041 Timisoara, Romania; 4 Department of Internal Medicine, “Victor Babes” University of Medicine and Pharmacy, 300041 Timisoara, Romania; 5 Department of Cardiology, Institute of Cardiovascular Diseases, 300020 Timisoara, Romania; 6 Department of Cardiology, Faculty of Medicine and Pharmacy, “Vasile Goldis” Western University of Arad, 310045 Arad, Romania; simonamercea19@gmail.com (S.M.); ppmldvn68@yahoo.com (A.P.M.); 7 Department of Neurology, Ovidius University of Constanta, 900527 Constanța, Romania; docuaxi@yahoo.com; 8 Deparment of Sport and Kinetotherapy, Faculty of Physical Education and Sport, Ovidius University of Constanta, 900527 Constanța, Romania; docuaxy@yahoo.com

**Keywords:** cardioembolic stroke, *MTHFR* gene polymorphisms, *C677T* mutation, *A1298C* mutation, non-valvular atrial fibrillation

## Abstract

Background: Cardioembolic stroke (CES), generally known as the most severe subtype of ischemic stroke, is related to many factors, including diabetes mellitus (DM), hypertension (HTN), smoking, hyperlipidemia and atrial fibrillation (AF). Genetic mutations of the methylenetetrahydrofolate reductase *(MTHFR)* gene *C677T* and *A1298C* have been recently associated with ischemic stroke. The purpose of this study was to analyze the prevalence of *MTHFR* gene polymorphisms correlated with cardiovascular risk factors in a selected population of patients with CES due to non-valvular AF (NVAF). Methods: This cross-sectional study was performed on 67 consecutive patients with acute cardioembolic stroke admitted to our hospital. The protocol included general physical examination, neurological clinical status and stroke severity evaluation, imagistic evaluation and genetic testing of *MTHFR* *C677T* and *A1298C* polymorphisms. Results: The prevalence of *MTHFR* polymorphisms in the study population was 38.2% for *C677T* and 40.3% for *A1298C*. The *C677T* mutation was significantly correlated with increased diastolic blood pressure (DBP) values (*p* = 0.007), higher total cholesterol (TC) (*p* = 0.003), low-density lipoprotein cholesterol (LDLc) (*p* = 0.003) and triglycerides (TGL) (*p* = 0.001), increased high-sensitive C-reactive protein (hsCRP) values (*p* = 0.015), HbA1c (*p* = 0.004) and left ventricle ejection fraction (LVEF) (*p* = 0.047) and lower high-density lipoprotein cholesterol (HDLc) (*p* < 0.001) compared to patients without this genetic variant. This genetic profile also included significantly higher CHA_2_DS_2_VASC (*p* = 0.029) and HASBLED (Hypertension, Abnormal liver/renal function, Stroke, Bleeding, Labile INR, Elderly age(>65 years), Drug/Alcohol usage history/Medication usage with bleeding predisposition) (*p* = 0.025) scores. Stroke severity in patients with *MTHFR* *A1298C* mutation was significantly increased when applying National Institutes of Health Stroke Scale (NIHSS) (*p* = 0.006) and modified Rankin scale (mRS) (*p* = 0.020) scores. The presence of *A1298C* mutation as a dependent variable was associated with significantly higher TGL values (odds ratio (OR) = 2.983, 95%CI = (1.972, 7.994)). Conclusions: The results obtained in this study demonstrate that *MTHFR* gene polymorphisms have a high prevalence in an NVAF cardioembolic stroke population. Moreover, an association between *C677T* mutation and stroke severity was highlighted. The *C677T* mutation in patients with NVAF was correlated with a higher incidence of cardiovascular comorbidities (hypertension HTN, heart failure (HF), dyslipidemia, type II diabetes mellitus (T2DM) with high HbA1c and increased inflammatory state). The *A1298C* *MTHFR* gene mutation was associated with a higher incidence of previous lacunar stroke and stroke recurrence rate, while dyslipidemia was the main cardiovascular comorbidity in this category.

## 1. Introduction

Ranked as the second leading cause of death worldwide, stroke causes an annual mortality rate of about 5.5 million. Moreover, in addition to high mortality, its high morbidity causes up to 50% of survivors to be chronically disabled. Thus, stroke is a disease of immense importance to public health, with serious economic and social consequences [1]. The most accurate definition of the stroke mechanism is very important, being the basis and guiding the most effective therapies and care [2].

About 14–30% of all cerebral infarctions are cardioembolic strokes (CES), generally the most severe subtype of ischemic stroke, with a low frequency of asymptomatic hospital discharge, a high risk of early and late embolic recurrences, and high mortality [3]. Despite the declining overall incidence of stroke, cardioembolic strokes have tripled in recent decades and could triple again by 2050 [2]. The incidence of CES increases with age [4]. Many factors, including diabetes mellitus (DM), hypertension (HTN), smoking, hyperlipidemia, atrial fibrillation (AF) and elevated plasma homocysteine levels (tHcy) are associated with an increased risk of this subtype of stroke [3,5]. Although all these risk factors play an important role in the occurrence of stroke, AF is still considered the leading cause of thromboembolism, being associated with cardioembolic strokes [6]. Strokes related to AF have higher mortality, higher disability rates, increased costs and increased incidence of recurrent stroke compared with non–AF-related strokes, making prevention of stroke due to nonvalvular atrial fibrillation (NVAF) a priority for physicians, patients and their families, as well as for society in general [7].

Elevated plasma homocysteine levels in particular are considered an independent risk factor, potentially modifiable, for cardioembolic stroke, this link being the object of numerous studies [5,8]. The most often-studied genetic variant, showing the strongest association with increased tHcy, is the cytosine (C) to thymine (T) substitution at position 677 in the methylenetetrahydrofolate reductase (*MTHFR*) gene (rs1801133) [9]. The *MTHFR* gene seems to be involved in lipid metabolism. A Chinese population-based study concluded that *MTHFR* polymorphisms could play an important role in the genetic determinism of serum lipid levels in hypertensive patients [10]. Selected gene polymorphisms were studied in association with lipid metabolism and the risk for ischemic stroke [11]. Another study found that the CYP2D6 mutation in the cytochrome P450 has an influence on the effects of lipid-lowering therapy in patients with hypercolesterolemia treated with simvastatin [12]. Munshi A. et al. studied the *1347G/A* mutation in the cytochrome P450 gene *CYP4F2* in hypertension and stroke. Their results indicated that this particular mutation is an important cardioembolic stroke risk factor [13].

The association of stroke with the *MTHFR C677T* variant showed inconsistent results in literature studies, most likely due to the small sample sizes and the different types of stroke studied [14]. A meta-analysis conducted by Holmes et al. that included 59,995 individuals from 237 databases of studies that reported possible associations between *MTHFR* polymorphisms, ischemic heart disease and stroke showed conflicting results [15]. Moreover, other authors consider that the association of *MTHFR C677T* genotype with ischemic stroke is only confined to the small cerebral vessel disease subtype [16]. Another fairly common mutation described in the *MTHFR* gene is the *A1298C* variant. This mutation is not associated with hyperhomocysteinemia (regardless of heterozygous or homozygous status), but a meta-analysis showed significant associations between *MTHFR A1298C* polymorphism and the risk of ischemic stroke in dominant and co-dominant inheritance models [17]. Moreover, Zhang et al. showed that *MTHFR* gene *A1298C* polymorphism plays a key role in the development of stroke. Furthermore, they highlight that *MTHFR A1298C* could increase the risk of stroke and may act as a predictor for clinical evolution [18].

Hyperhomocysteinemia has been suggested to play a role in NVAF pathogenesis [19]; however, the results from studies are controversial. Giusty et al., after studying 456 NVAF patients and 912 matched controls for *MTHFR C677T* and *A1298C* gene polymorphisms, observed a genotype–phenotype association between tHcy and *C677T MTHFR* polymorphism. The data demonstrated that the two polymorphisms, although able, at least in part, to affect tHcy, were not associated with an increased risk of NVAF per se or in combination [20]. The *A1298C MTHFR* gene mutation was also analyzed in 70 patients with NVAF and stroke and 70 healthy individuals with no documented episodes of AF matched for age, race and sex by Icli et al. The results of their study suggest that the *MTHFR* gene *A1298C* mutation appears not to be associated with non-valvular AF and ischemic stroke in the studied population [21].

In the present study, we selected both single nucleotide polymorphisms (SNPs) in the *MTHFR* gene: *C677T* and *A1298C*. The aim of this study was to determine their prevalence in cardioembolic stroke due to non-valvular atrial fibrillation and their correlations with cardiovascular risk factors, localization and severity of stroke.

## 2. Materials and Methods

### 2.1. Patient Population

From a total of 1565 patients with ischemic stroke admitted in the Neurology Department of Arad County Emergency Clinical Hospital, Romania, from January 2019 until March 2020, 70 consecutive patients with acute cardioembolic stroke were selected for this cross-sectional study. Three patients were excluded because they deceased before genetic testing was possible. All remaining patients included (*n* = 67) were previously diagnosed with non-valvular AF a minimum 5 years prior to current hospitalization and received oral anticoagulation therapy (Dicoumarins or NOAC). The presence of permanent atrial fibrillation was confirmed in all patients by previous 24 h Holter electrocardiogram (ECGs). Cardioembolic stroke was classified using the TOAST (Trial of Org 10,172 in Acute Stroke Treatment) classification according to the causative feature [22]. Patients with hemorrhagic conversion of CES were also included in the study and were classified according to ECASS (European Cooperative Acute Stroke Study) criteria into hemorrhagic infarction (without mass effect) and parenchymal hematoma (with mass effect) [23].

We classified CES according to the affected vascular territory: sylvian, carotidian and vertebrobasilar. Recurrent stroke and previous lacunar stroke were also analyzed as a further severity feature in affected patients and were diagnosed on imagistic studies. Patients with a history of previously documented comorbidities such as hypertension, coronary artery disease (CAD), peripheral artery disease (PAD), carotid atheromatosis, obesity, type II diabetes mellitus (T2DM) and dyslipidemia were included in the study. Patients with hyperthyroidism, systemic diseases, brain tumors or other oncologic pathologies and psychiatric disease such as psychosis and patients with insufficient clinical data were excluded from the study. None of the selected patients received thrombolysis therapy for acute stroke during their hospitalization. The study was approved by the Ethics Committee for Clinical Studies of the Arad County Emergency Clinical Hospital (according to the approval of the Ethics Committee for Clinical Research of the Arad County Emergency Clinical Hospital) with registration number 27/16.10.2019 and conformed to the Declaration of Helsinki. All patients included in the study gave their written informed consent for study enrollment.

### 2.2. Clinical and Biochemical Evaluation

The clinical evaluation included a general physical examination, evaluation of neurological clinical status, measurement of systolic and diastolic BP and the calculation of body mass index (BMI). Blood pressure (BP) was determined according to the European Guidelines on cardiovascular disease prevention in clinical practice [24]. In order to calculate the body mass index (BMI), body weight (kg) was determined with a mechanical scale and height (m) was measured using a metal talimeter (Fazzini, Vimodrone, Italy). BMI (kg/m^2^) was calculated according to the following formula: BMI = weight (kg) ÷ height^2^ (m^2^). An electrocardiogram was performed for every patient on admission using a 12-channel electrocardiograph ECG1212C (Contec Medical Systems, Qinhuangdao, Hebei Province, China) revealing atrial fibrillation.

Blood samples were collected for each patient on admission and the determinations were performed by the Medical Laboratory of Arad County Emergency Clinical Hospital. The lipid profile, respectively total cholesterol (TC), triglycerides (TGL), low-density lipoprotein cholesterol (LDLc) and high-density lipoprotein cholesterol (HDLc),) was determined using photometric methods (Dimension RXL-MAX, Dade Behring, Erlangen, Germany) for each patient enrolled in the study. HbA1c was determined with immunoturbidimetric assay, standardized by the Diabetes Control and Complications Trial (DCCT) [25] and certified by the National Glycohemoglobin Standardization Program (NGSP) [26]. The results were interpreted according to ADA recommendations: values between 4.8–5.6% as normal, 5.7–6.4% values representing a high risk for developing DM, and >6.5% as T2DM.

T2DM patients were diagnosed prior to hospital admission according to the consensus report of the American Diabetes Association (ADA) [27]. Other comorbidities such as chronic hypertension, CAD, PAD and HF (heart failure) were previously diagnosed according to the European Guidelines [24]. Heart failure was also classified according to the New York Heart Association (NYHA) in stages between I and IV [28].

High-sensitive C-reactive protein (hsCRP) was determined on an Abbott Architect C8000 platform (Abbott Diagnostics Headquarters, Illinois, USA) using CRP Vario (SENTINEL CH. SpA, Milano, Italy) reactant validated for Abbott platforms. The determination principle used was quantitative immunoturbidimetric, with a linearity method between 0.01–160 mg/L. Reference interval ≤5 mg/L was used for interpretation.

For the calculation of the cardioembolic risk, we assessed the CHA_2_DS_2_-VASC score, by using the following formula: congestive heart failure/LV dysfunction, hypertension, T2DM, vascular disease, age 65–75 years and female sex each representing 1 point, while the presence of stroke/transient ischemic attack (TIA) /thromboembolic events and age ≥75 years were noted each with 2 points summing up to a maximum of 9 points [29]. The HASBLED score was used in order to assess the bleeding risk, calculated with the following parameters: hypertension, abnormal renal/liver function, the presence of stroke, bleeding, labile international normalized ratio (INR), elderly age >65 years, drug/alcohol abuse/medication with bleeding predisposition each representing 1 point, with a maximum total of 7 points [30]. International normalized ratio (INR) was calculated from prothrombin time (determined using the coagulometer technique) with the following formula: INR = (prothrombin test/prothrombin control)^ISI^. INR values between 2–3 were considered as time in therapeutic range (TTR) for patients under dicoumarinic oral anticoagulants (OAC).

### 2.3. Evaluation of Stroke Severity

Stroke severity was evaluated on admission using the NIHSS (National Institutes of Health Stroke Scale) and modified Rankin scales. The NIHSS is a clinical scale used for the evaluation of consciousness, language, motor deficit, visual field loss, eye movements, ataxia and sensory loss, comprising a 15-item neurologic examination test. Each item is scored with 3, respectively 5 points and 0 is considered normal. Untestable items are also allowed since some clinical states imply the loss of certain cerebral functions. The interpretation of NIHSS is 0 for no stroke, 1–4 points for a minor stroke, 5–15 moderate stroke, 15–20 moderate to severe stroke and 21–41 for severe stroke [31]. The modified Rankin scale (mRS) is used for the evaluation of stroke disability with items scored 0 (no symptoms), 1 (no significant disability), 2 (slight disability), 3 (moderate disability), 4 (moderate to severe disability) and 5 (severe disability), and 6 representing death [32].

### 2.4. Imagistic Evaluation

All patients underwent imagistic evaluation by CT (computed tomography) in order to confirm the diagnosis, the localization and size of the cerebral lesion on admission. Cerebral CT scans were performed with CT Optima 520_16 slice serial no. 304758HMH6/2012 (General Electric Hangwei Medical Systems Co Ltd., Beijing, China).

### 2.5. Genetic Testing of MTHFR (C677T and A1298C) Polymorphisms

An amount of 3–6 mL of peripheral blood was collected from each patient on an EDTA vaccutainer. Genetic testing was performed at Medlife Laboratories, Bucharest, Romania. Genomic DNA was extracted using QIAamp DNA mini blood kit (Qiagen, Hilden, Germany). PCR amplification was made using LightCycler^®^ 480 Instrument II Platform (Roche Diagnostics GmbH, Mannheim, Germany) according to the manufacturer’s manual instructions. Hydrolysis probe assays for *MTHFR* gene polymorphisms identification were performed using TaqMan^®^ Master probes (Roche Diagnostics GmbH, Mannheim, Germany). This method implies using a single probe containing two oligonucleotide probes, respectively, a reporter dye (marked at the end of the 5′ nuclease with fluorophor) and a quencher (marked at the end of the 3′ nuclease). The quencher being close to the reporter as the probe is intact excites the fluorescence of the reporter via fluorescence resonance energy transfer (FRET). The resulted energy taken over by the quencher determines thermal relaxation. During PCR sequencing, the probe aligns with the recognition sequence, and the Taq polymerase hydrolyzes the probe during the amplification sequence. Increased fluorescent energy is accumulated during successive PCR cycles and then measured. For this method, 5 fluorophores were used to mark the probes, respectively, FAM, HEX, TexasRed and Cy5. The quenchers used in the process were BHQ1 and BHQ2 (Black Hole Quenchers). In order to detect *C677T* polymorphism FAM and HEX marked probes were used. Wild-type sequence is recognized by the FAM marked probe, while HEX marked probe detects the polymorphism (mutant) sequence. For *A1298C* polymorphism TexasRed (for wild type) and Cy5 (for polymorphism) marked probes were used. At the end of the process, the results were analyzed with the genotyping module of LightCycler^®^ software version 1.5 (Roche Diagnostics Corporation P.O., Indianapolis, IN, USA).

### 2.6. Ultrasound Evaluation of the Heart and Carotid Arteries

Transthoracic echocardiography was performed with Acuson SC2000 Prime ultrasound system, manufactured by Siemens Healthineers (Siemens Healthcare GmbH, Erlangen, Germany). A standard echocardiographic evaluation was performed in each patient included in the study. With the use of ultrasound 2D-mode, we determined the dimensions of the heart cavities and walls and the presence of valvular lesions, calculated the ejection fraction and quantified systolic and diastolic functions. Spectral and color Doppler ultrasound by two variants was used to quantify valvular regurgitation, systolic and diastolic functions of the left ventricle (LV) and pressures in the pulmonary circulation. Patients with valvular diseases were excluded from the study. For this study, we selected only 3 echocardiographic parameters for statistical analysis, respectively left atrium volume (LAV), left ventricle ejection fraction (LVEF) and left ventricle end-diastolic volume (LVEDV). Left atrium volume (LAV) was measured during end-systole just before mitral valve opening also from standard apical 4-chamber view. Using planimetry, we determined left atrium (LA) borders by respecting the walls of the left atrium and excluding pulmonary veins and left atrial appendage. LAV was selected for the predictive value of this parameter to quantify LA structural anatomical changes due to NVAF. While AF can lead to heart failure with preserved EF and normal systolic function, LVEDV was selected as the most suitable parameter to determine the left ventricle damage caused by AF [33]. LVEF was calculated according to Simpson’s formula as a percentage of change in volumes between diastole and systole: EDV-ESV/EDV × 100 by using apical 4-chamber view.

Doppler carotid echography was performed in all patients included in the study. In order to detect carotid atheromatosis, we performed intima-media thickness (IMT) measurements, with values of <0.9 mm considered as normal. The absence/presence of carotid plaques was also quantified.

### 2.7. Statistical Analysis

Statistical processing was performed in SPSSv17 (version 17, SPSS Inc., Chicago, IL, USA) and Microsoft Excel (version 2013, MS Corp., Redmond, WA, USA). In the case of nominal variables, the frequency tables were prepared together with the “pie” type graphs. The associations between the dichotomic variables and other variables were made with the logistic regression method, and comparisons between these types of variables were made with the Chi^2^ test. The risk analysis was done by interpreting the odds ratio (OR) and the 95% confidence interval for the OR. For the numerical variables, the mean values, standard deviations and standard error of the mean were calculated, and boxplot and column graphs were made. Comparisons between numerical series were performed with nonparametric Mann-Whitney tests in case of comparisons between two series of values with non-Gaussian distribution. We used the value of *p* <0.05 for significant differences. Logistic regression according to the Cox and Snell R-square model and multivariate linear regression (Enter method) were used to identify all potential predictors for cardioembolic stroke. For multiple comparisons we applied Kruskal–Wallis test using a value of *p* <0.001 for significant differences.

## 3. Results

Patient characteristics including demographic, clinical, biochemical and echographic data for comparisons between presence and absence of *MTHFR C677T* mutation are summarized in Table 1.

By comparing patients with (*n* = 26) vs. without (*n* = 41) *MTHFR C677T* mutation, significantly increased values for DBP (88.9 ± 11.593 mmHg vs.81.5 ± 8.846 mmHg, *p* = 0.007), TC (176.96 ± 46.64 mg/dL vs. 218.34 ± 59.52 mg/dL, *p* = 0.003), LDLc (152.02 ± 52.85 mg/dL vs. 115.69 ± 33.83 mg/dL, *p* = 0.003), HbA1c (6.23 ± 1.14% vs. 5.53 ± 1.02%, *p* = 0.004), TGL (266.1 ± 149.3 mg/dL vs. 114.12 ± 52.24 mg/dL, *p* < 0.001), LVEF (50.42 ± 11.06 mL vs. 45.37 ± 9.94 mL, *p* = 0.047) and hsCRP (10.4 ± 5.81 mg/L vs. 7.01 ± 4.98 mg/L, *p* = 0.015) were found in the *C677T* mutation group. In addition, values for NIHSS (*p* = 0.001), mRS (*p* = 0.003), CHA_2_DS_2_VASC (*p* = 0.029) and HASBLED (*p* = 0.025) scores were significantly increased in this group of patients. HDLc values were significantly decreased in patients with *C677T* mutation (51.31 ± 14.92 mg/dL vs. 36.24 ± 13.92 mg/dL, *p* < 0.001). Patients with *C677T* had significantly increased HT grades (*p* = 0.037) and HF stages (*p* = 0.016) compared to patients without this *MTHFR* gene mutation. There were no significant differences between groups regarding age (*p* = 0.119), BMI (*p* = 0.054), SBP (*p* = 0.054), LAV (*p* = 0.252), LVEDV (*p* = 0.055), IMT (*p* = 0.172) and INR values (*p* = 0.638) (Table 1).

Regarding cardiovascular comorbidities, there were no significant differences between groups for carotid atheromatosis (*p* = 0.660), CAD (*p* = 0.138), PAD (*p* = 0.051) and T2DM (*p* = 0.804) (Table 2). There were also no significant differences between groups regarding the male sex, as an additional cardiovascular risk factor (*p* = 0.124). However, by using Chi^2^ tests, the proportion of patients with ischemic CES in the sylvian artery territory was significantly higher in the non-mutant group (95.1% vs. 61.5%, *p* = 0.002), while the proportion of vertebrobasilar CES (23.1% vs. 2.4%, *p* = 0.002), hemorrhagic conversion of CES, respectively the hemorrhagic infarction subtype (15,4% vs. 0%, *p* = 0.020) and previous lacunar stroke (57.7% vs. 19,5%, *p* = 0.003) was significantly higher in patients with *C677T* mutation. Our results also hinted that the proportion of patients with recurrent stroke was significantly higher in the *C677T* mutation group (69.2% vs.24.4%, *p* < 0.001) (Table 2).

The comparison between patients with (*n* = 27) vs. patients without (*n* = 40) *MTHFR A1298C* mutation revealed no significant differences between groups regarding age (*p* = 0.192), BMI (*p* = 0.194), CHA2DS2-VASC scores (*p* = 0.534), HASBLED score (*p* = 0.090), HTN grades (*p* = 0.173), HF stages (*p* = 0.070), DBP (*p* = 0.408), SBP (*p* = 0.356), HbA1c (*p* = 0.111), LAV (*p* = 0.636), LVEDV (0.328), LVEF (*p* = 0.552), IMT (*p* = 0.091), hsCRP (*p* = 0.385) and INR (*p* = 0.263) (Table 3).

In patients with *MTHFR A1298C* mutation, stroke severity was significantly increased with higher NIHSS (*p* = 0.006) and mRS (*p* = 0.020) scores. The values for the lipidic profile of patients with *A1298C* mutation were significantly higher vs. patients without this mutation: TC (222 ± 60.09 mg/dL vs. 173.07 ± 41.09 mg/dL, *p* = 0.001), LDLc (158.78 ± 43.22 mg/dL vs. 107.04 ± 41.76 mg/dL, *p* < 0.001) and TGL (268.83 ± 146.58 mg/dL vs.115.7 ± 65.45 mg/dL, *p* < 0.001). HDLc values were significantly lower in patients with *A1298C* mutation (36.9 ± 13.43 mg/dL vs. 49.78 ± 16.65 mg/dL, *p* = 0.001) (Table 3).

There were no significant differences between patients with *A1298C MTHFR* mutation vs. without mutation regarding gender (*p* = 0.318), the presence of carotid atheromatosis (*p* = 0.886), CAD (*p* = 0.459), PAD (*p* = 0.059) and T2DM (*p* = 0.618). When we compared the proportion of patients according to arterial localization of CES, we found no significant differences between groups for sylvian ischemic CES and vertebrobasilar ischemic CES (*p* = 0.161). Moreover, the difference between the proportion of patients with hemorrhagic conversion of CES, respectively hemorrhagic infarction (*p* = 0.295) and parenchymal hematoma (*p* = 0.403) was statistically insignificant between the two groups. However, significant differences were found in patients with *A1298C* mutation regarding the proportion of previous lacunar stroke lesions (51.9% vs. 22.5%, *p* = 0.019) and the presence of recurrent stroke (59.3% vs. 30%, *p* = 0.024) (Table 4).

Logistic regression was performed using the Forward Stepwise (Wald) method considering the *C677T* mutation in the *MTHFR* gene as a dependent variable. Cox and Snell R Square result was 0.519, and variables such as DBP, HbA1c and TGL were significantly associated with the *MTHFR C677T* mutation in both homozygotes and heterozygotes (Table 5).

**Figure 1:**
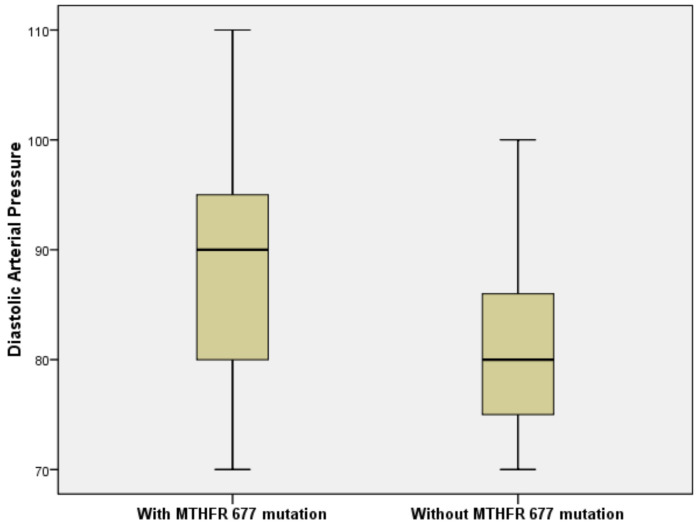
Patients with *C677T* mutation have a higher risk for developing higher DBP values (odds ratio (OR) = 1.881, 95%CI = (1.804, 4.964)).

DBP values were significantly higher in patients with *MTHFR C677T* mutation compared to patients without this mutation (Mann–Whitney U Test, *p* = 0.007). Patients with *C677T* mutation also had a higher risk for developing high DBP values (OR = 1.881, 95% CI= (1.804, 4.964)) (Figure 1).

**Figure 2:**
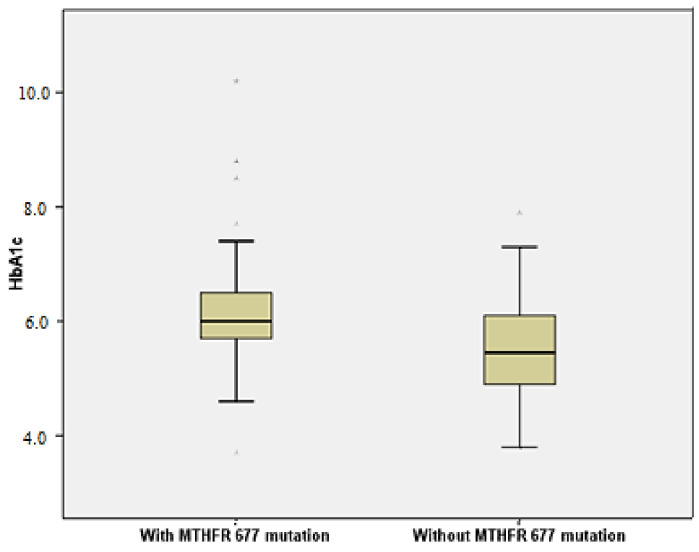
Patients with *MTHFR C677T* mutation have a high risk for increased HbA1c values (OR = 1.982, 95%CI = (1.171, 7.799)).

**Figure 3:**
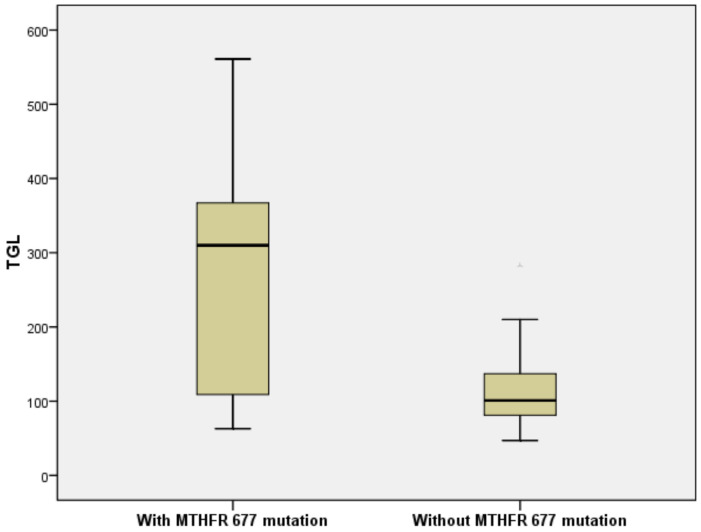
*C677T* mutation in the *MTHFR* gene is associated with a high risk for hypertrygliceridemia (OR = 1.392, 95%CI = (1.192, 3.994)).

Patients with *C677T* mutation had a higher risk for increased HbA1c values (OR = 1.982, 95%CI= (1.171, 7.799)). Mann–Whitney U test also revealed that the *C677T* mutation in the *MTHFR* gene was associated with significantly higher HbA1c values vs. without mutation (*p* = 0.004) (Figure 2). TGL values were also significantly higher in the *C677T* mutation group compared to the group without the mutation (Mann–Whitney U Test, *p* < 0.001). Patients with *C677T* polymorphism also had a high risk for hypertriglyceridemia (OR = 1.392, 95%CI = (1.192, 3.994)) (Figure 3).

**Figure 4:**
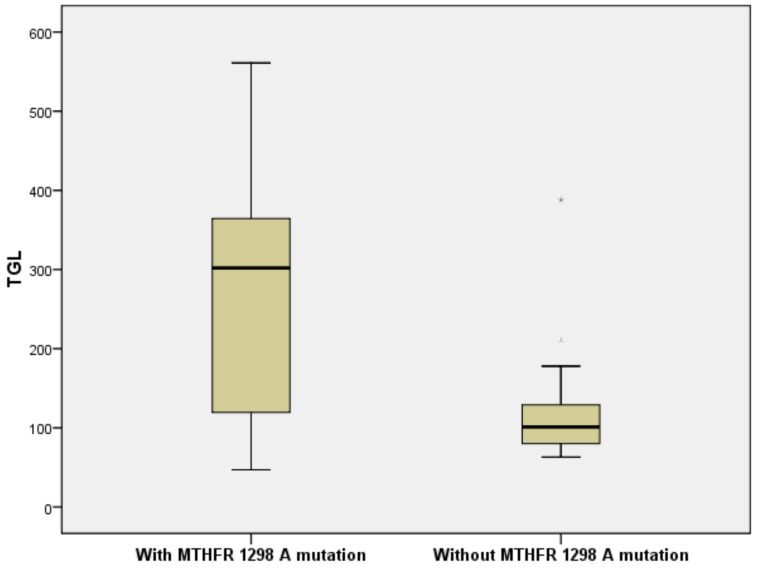
*MTHFR A1298C* is associated with a risk for high triglycerides (TGL) values (OR = 2.983, 95%CI = (1.972, 7.994)).

Logistic regression performed using the *MTHFR A1298C* mutation as a dependent variable (Forward Stepwise (Wald) method) (Table 6) revealed that patients with the *A1298C* mutation had a risk for developing high TGL values (OR = 2.983, 95%CI = (1.972, 7.994)) (Figure 4). Significantly higher TGL values were found in patients with *A1298C* mutation compared to the non-mutant group (Mann–Whitney U Test, *p* < 0.001).

**Figure 5:**
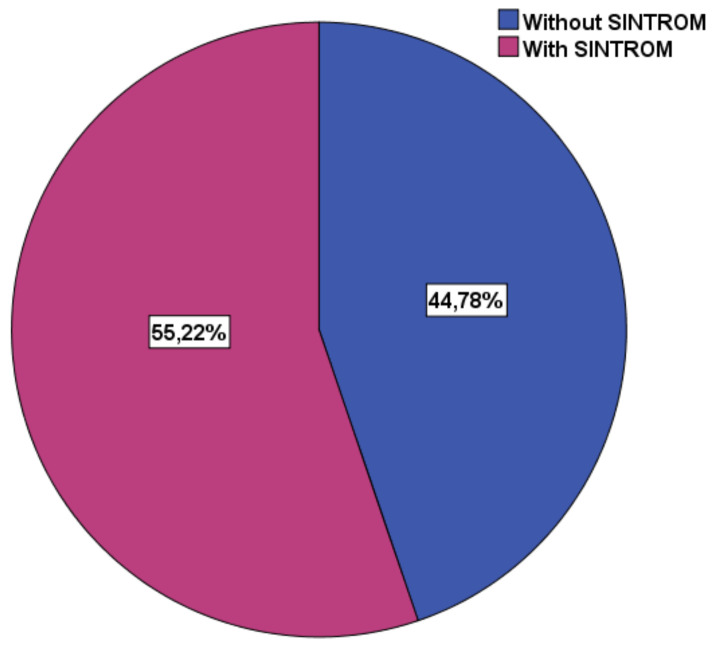
Percentage of patients on acenocumarol vs. NOAC.

Mean INR was 1.82 ± 0.49, revealing insufficient anticoagulant effect in the 37 patients (55.22%) with NVAF on acenocumarol (Table 7). The rest of the 30 patients (44.78%) were on novel oral anticoagulants (NOAC) (Figure 5).

**Figure 6:**
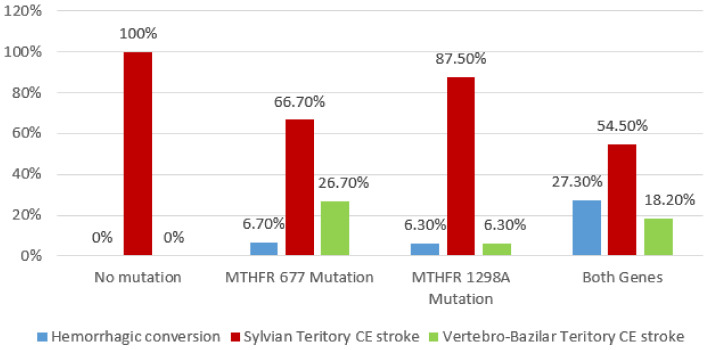
Association between cardioembolic stroke localization and *MTHFR* polymorphism by applying Chi^2^ Test.

The multiple comparison analysis using Chi^2^ test revealed a significant association between CES stroke localization and *MTHFR* gene polymorphisms (*p* = 0.008) (Figure 6).

**Figure 7:**
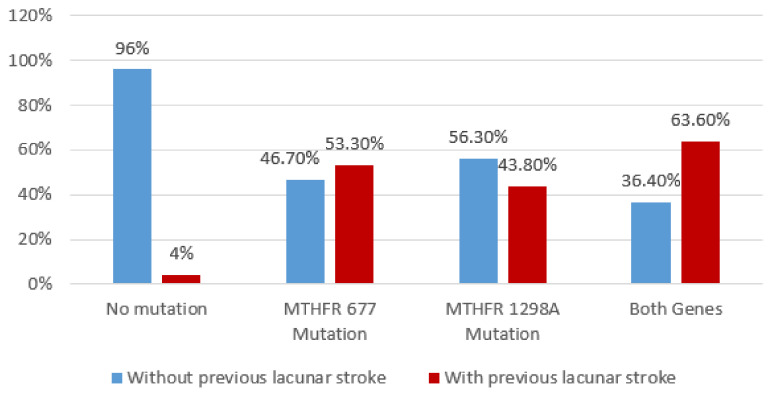
Association between previous lacunar stroke and *MTHFR* polymorphisms using Chi^2^ Test.

**Figure 8:**
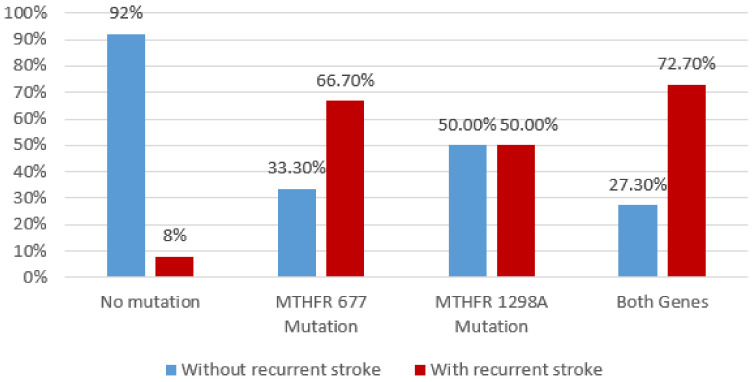
Association between recurrent stroke and *MTHFR* polymorphisms by Chi^2^ Test.

By using multiple comparison analysis, our results showed a significant association between previous lacunar stroke (*p* = 0.001, Chi^2^ Test), respectively, recurrent stroke with *MTHFR* polymorphisms (Chi^2^ Test, *p* < 0.001) (Figure 7 and Figure 8).

**Figure 9:**
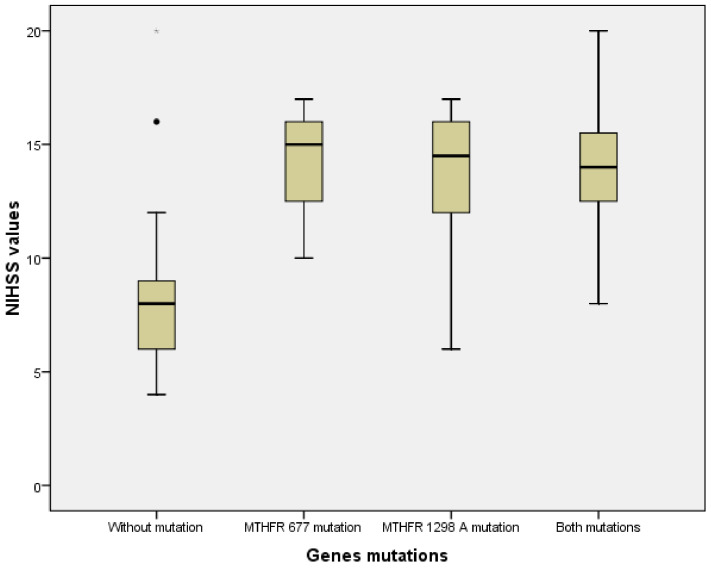
Comparison of NIHSS scores between patients without vs. with one or both *MTHFR* polymorphisms.

NIHSS scores were significantly lower for patients without *MTHFR* polymorphisms when compared to patients with one or both genetic variants (Kruskal–Wallis test, *p* < 0.001) (Figure 9).

**Figure 10:**
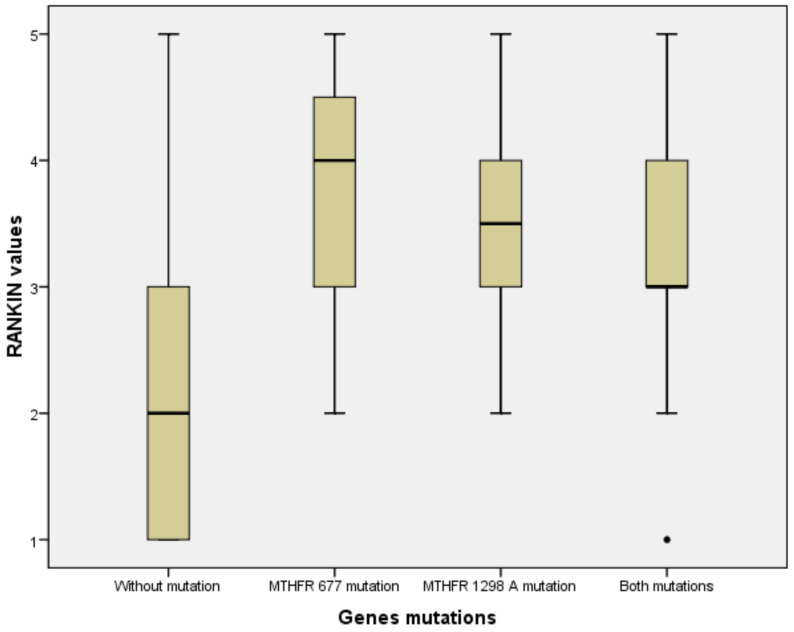
Comparison between patients without vs. with one or both polymorphisms of the *MTHFR* gene regarding mRS scores.

Stroke severity was also analyzed by mRS scores between patients without SNPs of *MTHFR*, with one (*C677T*, respectively *A1298C*) and patients with both SNPs using the Kruskal–Wallis test. The mRS scores were significantly lower for patients without *MTHFR* polymorphisms (*p* < 0.001) (Figure 10).

When comparing HbA1c, TC, LDLc and TGL values between *MTHFR* groups, we found significantly increased values in patients with both *MTHFR* SNPs vs. patients with one or none SNP (*p* = 0.009 for HbA_1_c, respectively *p* < 0.001 for TC, TGL and LDLc). HDLc values were significantly higher in patients without *MTHFR* mutations (*p* < 0.001). We found no significant differences for SBP (*p* = 0.144), DBP (*p* = 0.074), LVEF (*p* = 0.176) and IMT (*p* = 0.147) between *MTHFR* groups.

## 4. Discussion

The genetic variants of the *MTHFR* gene, respectively *C677T* and *A1298C* mutations, have been studied by numerous authors over time. Although these genetic mutations have proven to be inherited [34] and associated with several clinical conditions [35], there is a lack of clinical studies regarding the connection between these mutations and cardioembolic stroke. A meta-analysis performed by Kang S. et al. discovered a link between the high ischemic stroke risk and the presence of the *A1298C* polymorphism [17]. Our study aimed to look for the associations between genetic variants of the *MTHFR* gene in CE stroke patient, localization and clinical severity of strokes, and also additional cardiovascular risk factors involved in NVAF.

Although the American College of Medical Genetics and Genomics guidelines recommended in 2013 against routine *MTHFR* genetic testing in cardiovascular diseases [36], a study conducted by Biselli et al. has suggested a strong association between coronary artery disease incidence and *MTHFR A1298C* mutation [37]. Meng et al. [38] and Zhu et al. [39] already tried to analyze the association between this mutation in the *MTHFR* gene and T2DM, but the concomitant presence of cardioembolic ischemic stroke was not included in their analysis. Another study by Hermans MP et al. [40] demonstrated on a relatively small cohort (165 patients) the link between *C677T* polymorphism (both homozygous and heterozygous) and the risk for ischemic stroke in patients with T2DM. Moreover, they suggest that the *MTHFR C677T* mutation confers a higher risk for stroke to both homozygous and heterozygous T allele carriers. The impact of the *MTHFR* polymorphism on stroke may result from T allele-linked deleterious effects or C allele-linked protection; however, the authors consider that more studies are needed to support this hypothesis [40].

Numerous studies regarding inflammation in stroke have been conducted so far, but Chen et al. managed to find the associations between neuroinflammation due to overrated microglial activation, augmented by elevated plasma levels of tHcy in the middle cerebral artery occlusion (*MCAO*) in a rat model with ischemic stroke [41]. Our study population with *C677T* mutation was predisposed to have more cardiovascular risk factors with significantly increased DBP values (*p* = 0.007), dyslipidemia with high TC (*p* = 0.003), LDLc (*p* = 0.003) and TGL (*p* = 0.001), while HDLc was significantly lower (*p* < 0.001) compared to patients without this genetic variant. Significantly increased HbA1c (*p* = 0.004) and high hsCRP values (*p* = 0.015) were also found in these patients. The general profile of the patients with *C677T* mutation in our study included significantly higher CHA2DS2VASC (*p* = 0.029) and HASBLED (*p* = 0.025) scores, leading to the conclusion that this category is at higher risk for thromboembolic events. Hu X. et al. also studied the connection between five single-nucleotide polymorphisms related to ischemic stroke (including *MTHFR C677T* mutation) and CES in NVAF. Their results demonstrated that high fibrinogen plasma levels due to the β-fibrinogen gene 455 G/A mutations are independently associated with CES risk in patients with decreased CHA2DS2-VASC scores [42].

Stroke severity in patients with the *C677T* mutation was also higher in patients with increased NIHSS (*p* = 0.001) and mRS (*p* = 0.003) scores in our study. A Chinese-based population study was carried out in 2005 and demonstrated an important connection between stroke severity (high NIHSS scores) and the perturbance of the *MTHFR* genetic activity (A222V amino acid change mutation, which further increases homocysteine levels), but their study did not refer to specific genetic variants of the *MTHFR* gene [43]. Our studied patients with *C677T* mutation were also more likely to have severe cardiovascular comorbidities with significantly increased HT grades (*p* = 0.037) and HF stages (*p* = 0.016). Arsene et al. performed a study in 2011 but failed to demonstrate an association between *MTHFR* polymorphisms and ischemic stroke, mainly due to their small sample size (67 patients and 60 controls). However, their studied population had a high incidence in hypertension and dyslipidemia (TGL and cholesterol) in patients with ischemic stroke [14].

We also analyzed the connection between the presence of the *C677T* mutation and stroke lesion localization on the premise that the affected cerebral area is important for the clinical severity at presentation. Vertebrobasilar CES (*p* = 0.02), hemorrhagic infarction subtype of hemorrhagic conversion of CES (*p* = 0.020) and previous lacunar stroke (*p* = 0.003) were more frequent in the *C677T* mutation group. Patients with this genetic mutation were also more likely to have recurrent stroke (*p* < 0.001) as a consequence of the high thrombotic state and additional cardiovascular risk factors.

Cardioembolic strokes due to the severity of arterial occlusion by a large thrombus often cause hemorrhagic conversion of an initially ischemic stroke. A systematic review on cardioembolic stroke by Arboix et al. stated that hemorrhagic conversion of CES occurs in approximately 71% of cases. The same authors also found secondary hematomas in a small proportion of patients, respectively 0.8%. Although they do not occur with the same frequency as the hemorrhagic transformation, they are found in patients with NVAF and CES [44]. Several studies tried to identify an association between *MTHFR* genetic variants and stroke. Kang S et al. performed a meta-analysis in 2013 and found that the *C677T* mutation was associated with a high risk for hemorrhagic stroke, especially the T allele [45]. Other authors demonstrated that the two genetic *MTHFR* variants are independent genetic risk factors for stroke, both ischemic and hemorrhagic [46]. A study on an Indian population also indicated the *C677T* mutation as an important stroke risk factor but failed to make a distinction between ischemic and hemorrhagic stroke [47].

A study conducted by Rutten-Jacobs LC et al. managed to find an association between small-vessel cerebral infarctions and the presence of *C677T* gene mutation in the studied population [16]. Soriente et al. observed that *C677T* gene mutation is associated with stroke severity; however, they suggest that combined data are consistent with the possibility that genotype-based observations can be applied only to ethnic groups of similar genetic backgrounds [48].

Our results also showed a significantly elevated lipidic profile, i.e., TC (*p* = 0.001), LDLc (*p* < 0.001) and TGL (*p* < 0.001) in patients with *A1298C* mutation with significantly lower HDLc values (*p* = 0.001) compared to non-mutation.

In a study on 84 patients with ischemic stroke compared with 100 healthy controls where the most common stroke risk factors were investigated, tHcy levels were found to be associated with the *MTHFR C677T* variant; however, no significant association was found with the *MTHFR A1298C* variant [49]. In our patients with *MTHFR A1298C* mutation, stroke severity was significantly increased with higher NIHSS (*p* = 0.006) and mRS (*p* = 0.020) scores. Patients with *A1298C* mutation were more likely to have previous lacunar stroke lesions (*p* = 0.019), and recurrent stroke was also more frequent (*p* = 0.024) compared to non-mutation.

When analyzing the presence of the *C677T* mutation as a dependent variable (logistic regression, Cox and Snell R Square = 0.519), variables such as DBP, HbA1c and TGL have proven to be significant predictors for the *MTHFR C677T* mutation for both homozygotes and heterozygotes. Cardiovascular risk factors such as increased DBP (OR = 1.881, 95% CI= (1.804, 4.964)), hypertriglyceridemia (OR = 1.392, 95%CI= (1.192, 3.994)) and elevated HbA1c (OR = 1.982, 95%CI = (1.171, 7.79)) were important risk factors associated with the *C677T* mutation in CES due to non-valvular AF in our study. In a study regarding cardiovascular risk factors in patients with ischemic stroke, no significant differences were found in age, sex, *MTHFR C677T* mutation status, total homocysteine levels or other biochemical parameters between patients with lacunar stroke and non-lacunar stroke. However, the history of hypertension and smoking was significantly higher in patients with lacunar infarction (*p* = 0.001 and 0.02, respectively) [50].

For the *A1298C* mutation analyzed as a dependent variable, we found an association only with high TGL values (OR = 2.983, 95%CI = (1.972, 7.994)). The *A1298C MTHFR* gene mutation was analyzed by Icil et al. in a study on 70 patients with NVAF and stroke and 70 healthy individuals with no documented episode of AF matched for age, race and sex. Their results showed no significant difference for age and gender for the presence of the *A1298C MTHFR* gene mutation between groups [21]. Until now, there were no reported studies which analyzed cardiovascular risk factors and both *C677T* and *A1298C MTHFR* gene mutations in patients with cardioembolic stroke, supporting the uniqueness of the current study.

The enrolled NVAF patients on acenocumarol therapy in our study also had a higher risk for thromboembolic events due to the low INR values (mean 1.82 ± 0.49). Studying the new oral anti-coagulants versus acenocumarol in high thromboembolic risk patients with NVAF, Bellin et al. observed that NOAC treatment proved to be superior to acenocumarol, leading to less cardiovascular events and less bleeding episodes (*p* < 0.0001 and *p* = 0.0049, respectively) [51].

CE stroke localization according to affected arterial territory and hemorrhagic conversion of CES were significantly associated with *MTHFR* polymorphisms at multiple comparison analysis using Chi^2^ tests (*p* = 0.008, respectively *p* = 0.009). *MTHFR* SNPs were also significantly associated with previous lacunar stroke (*p* = 0.001) and recurrent stroke (*p* < 0.001) in our study population. Regarding stroke severity, NIHSS and modified Rankin scale scores were significantly lower in patients without *MTHFR* mutations (*p* < 0.001 in both associations). Patients with both genetic variants of *MTHFR* had significantly increased HbA_1_c (*p* = 0.009), TC, LDLc and TGL (*p* < 0.001) values compared with patients with only a single mutation and with no mutation in the *MTHFR* gene.

Study limitations: In this study, we only analyzed the prevalence of mutations within the selected stroke population. Due to budget limitations, we did not include a non-stroke control group. In this all-case design, the association between the *MTHFR* polymorphisms and risk factors such as blood lipids and T2DM were examined and significant associations were found. The comparisons were made between mutation vs. non-mutation groups. However, as the analysis was restricted to stroke cases, lacking a non-stroke control group, our results could be commented as “collider bias”, leading to a false positive association between the SNPs and risk factors. A purported bias can be due to confounding or selection in many real-data settings, but the distinction may not matter [52].This potential bias could be true for most of the other phenotypes that have been evaluated in previous studies. The relatively small sample size and the lack of plasma tHcy levels determination are other limitations of this study.

## 5. Conclusions

Our study demonstrated the prevalence of *MTHFR* gene polymorphisms in a cardioembolic stroke population with NVAF. Moreover, it highlighted the relationship between *C677T* mutation and stroke lesion type and localization (especially vertebrobasilar CES, hemorrhagic conversion of CES and previous lacunar stroke), but also with recurrent stroke lesions and stroke severity (high NIHSS and mRS scores). The *C677T* mutation in patients with NVAF was associated with a higher incidence of cardiovascular comorbidities (HTN, HF, dyslipidemia, T2DM with high HbA1c and increased inflammatory state), thromboembolic and bleeding risk compared to patients without this genetic variant. The *A1298C* mutation was also associated with a higher previous lacunar stroke incidence and stroke recurrence rate, while only dyslipidemia was the main cardiovascular comorbidity. Cardiovascular risk factors such as DBP, hypertriglyceridemia and elevated HbA1c were important risk factors associated with the *C677T* mutation, while only hypertriglyceridemia was associated with the *A1298C* mutation. Larger cohort studies are necessary to confirm the implications of these two *MTHFR* genetic variants, and to study their further role in genetic therapies in patients with high thrombotic states at risk for cardioembolic stroke.

## Figures and Tables

**Table 1:** Variable comparisons for *MTHFR C677T* mutation (*n* = 67) in all patients using the Mann–Whitney U Test.

Variable	Without *MTHFR C677T* Mutation (*n* = 41)		With *MTHFR C677T* Mutation (*n* = 26)		*p* ^T^
	Mean ± Std. Deviation (Median)	Mean Rank	Mean ± Std. Deviation (Median)	Mean Rank	
Age, y	73.00 ± 8.09	31.05	75.00 ± 9.03	38.65	0.119
BMI, kg/m^2^	30.3 ± 4.04	28.25	32.01 ± 3.25	37.65	0.054
NIHSS	(9)	27.66	(15)	44.00	0.001 ^*^
mRS	(3)	28.52	(4)	42.63	0.003 ^*^
CHA_2_DS_2_VASC	(5)	29.93	(7)	40.42	0.029 ^*^
HASBLED	(3)	29.96	(4)	40.37	0.025 ^*^
HTN grade, *n* (%)	(2)	28.90	(2)	37.23	0.037 ^*^
HF NYHA, *n* (%)	(0)	27.21	(2)	38.30	0.016 ^*^
DBP, mmHg	81.5 ± 8.846	25.98	88.9 ± 11.593	39.09	0.007 ^*^
SBP, mmHg	159.19 ± 18.88	28.27	168.49 ± 23.57	37.63	0.054
TC, mg/dL	218.34 ± 59.52	39.66	176.96 ± 46.64	25.08	0.003 ^*^
LDLc, mg/dL	115.69 ± 33.83	25.19	152.02 ± 52.85	39.59	0.003 ^*^
HDLc, mg/dL	51.31 ± 14.92	46.27	36.24 ± 13.92	26.22	<0.001 ^*^
HbA1c,%	5.53 ± 1.02	25.40	6.23 ± 1.14	39.45	0.004 ^*^
TGL, mg/dL	114.12 ± 52.24	22.02	266.1 ± 149.3	41.60	<0.001 ^*^
LAV, mL	79.8 ± 29.25	31.83	85.19 ± 22.41	37.42	0.252
LVEDV, mL	122.56 ± 34.97	30.37	135.5 ± 31.19	39.73	0.055
LVEF,%	45.37 ± 9.94	30.24	50.42 ± 11.06	39.92	0.047 ^*^
IMT, mm	0.92 ± 0.3	31.43	1.03 ± 0.28	38.06	0.172
hsCRP, mg/L	7.01 ± 4.98	29.38	10.4 ± 5.81	41.29	0.015 ^*^
INR	1.76 ± 0.52	17.93	1.85 ± 0.49	19.65	0.638

^T^—Mann-Whitney U Test; *—significant difference. BMI, body mass index; NIHSS, National Institutes of Health Stroke Scale; mRS, modified Rankin Scale; CHA_2_DS_2_-VASC, acronym for Congestive Heart Failure, Hypertension, Age (>75 years), Diabetes Mellitus, Stroke/TIA (transient ischemic attack)/TE (thromboembolism), Vascular disease, Age (65–74 years), Sex category; HTN, hypertension; HASBLED, acronym for Hypertension, Abnormal liver/renal function, Stroke, Bleeding, Labile INR, Elderly age (>65 years), Drug/Alcohol usage history/Medication usage with bleeding predisposition; HF NYHA, heart failure New York Heart Association functional classification; DBP, diastolic blood pressure; SBP, systolic blood pressure; TC, total cholesterol; LDLc, low-density lipoprotein cholesterol; HDL-c, high-density lipoprotein cholesterol; HbA1c, glycosylated hemoglobin; TG, triglycerides LAV, left atrial volume; LVEDV, left ventricular end-diastolic volume; LVEF, left ventricular ejection fraction; IMT, intima media thickness; hsCRP, high sensitive C-reactive protein; INR, International Normalized Ratio. Values were expressed as mean ± standard deviation (SD).

**Table 2:** Variable comparisons regarding of cardiovascular comorbidities and stroke localization. for *MTHFR C677T* mutation (*n* = 67) using the Chi^2^ Test.

Variable	Without *MTHFR C677T* Mutation (*n* = 41)	With *MTHFR C677T* Mutation (*n* = 26)	*p* ^T^
	*n* (%)	*n* (%)	
Sex (male)	20 (48.8%)	7 (26.9%)	0.124
Carotid Atheromatosis	23 (56.1%)	16 (61.5%)	0.660
CAD	34 (82.9%)	25 (96.2%)	0.138
PAD	12 (29.3%)	1 (3.8%)	0.051
T2DM	16 (39.0%)	11 (42.3%)	0.804
Ischemic CES:			0.002 ^*^
Sylvian territory	39 (95.1%)	16 (61.5%)	
Vertebrobasilar territory	1 (2.4%)	6 (23.1%)	
Hemorrhagic conversion of CES:			
Hemorrhagic infarction	0 (0%)	4 (15.4%)	0.020 ^*^
Parenchymal hematoma	1 (2.4%)	0(0%)	0.612
Previous lacunar stroke	8 (19.5%)	15 (57.7%)	0.003 ^*^
Recurrent stroke	10 (24.4%)	18 (69.2%)	<0.001 ^*^

^T^—Chi^2^ Test *—significant difference. CAD, coronary artery disease; PAD, peripheral vascular disease; T2DM, type II diabetes mellitus; CES, cardioembolic stroke.

**Table 3:** Variable comparisons for *MTHFR A1298C* mutation (*n* = 67) in all patients using the Mann–Whitney U Test.

Variable	Without *MTHFR A1298C* Mutation (*n* = 40)		With *MTHFR A1298C* Mutation (*n* = 27)		*p* ^T^
	Mean + Std. Deviation (Median)	Mean Rank	Mean + Std. Deviation (Median)	Mean Rank	
Age, y	72.38 ± 8.43	31.45	75.85 ± 8.21	37.78	0.192
BMI, kg/m^2^	30.95 ± 3.99	31.46	31.95 ± 3.04	37.76	0.194
NIHSS	(10)	28.61	(14)	41.98	0.006 ^*^
mRS	(3)	29.58	(3)	40.56	0.020 ^*^
CHA_2_DS_2_VASC	(6)	32.80	(6)	35.78	0.534
HASBLED	(4)	30.85	(4)	38.67	0.090
HTN grade, *n*(%)	(2)	30.78	(2)	36.18	0.173
HF NYHA, *n*(%)	(0)	29.04	(2)	37.35	0.070
DBP, mmHg	84.85 ± 11.64	31.63	86.83 ± 10.88	35.60	0.408
SBP, mmHg	162.73 ± 23.13	32.20	168.07 ± 20.74	36.67	0.356
TC, mg/dL	173.07 ± 41.09	24.59	222 ± 60.09	40.35	0.001 ^*^
LDLc, mg/dL	107.04 ± 41.76	21.63	158.78 ± 43.22	42.35	<0.001 ^*^
HDLc, mg/dL	49.78 ± 16.65	43.54	36.9 ± 13.43	27.56	0.001 ^*^
HbA1c,%	5.83 ± 1.47	29.39	6.05 ± 0.86	37.11	0.111
TGL, mg/dL	115.7 ± 65.45	22.06	268.83 ± 146.58	42.06	<0.001 ^*^
LAV, mL	80.37 ± 26.33	33.08	84.15 ± 27.72	35.37	0.636
LVEDV, mL	123.84 ± 30.64	32.09	133.13 ± 38.19	36.83	0.328
LVEF, mL	45.7 ± 12.48	32.28	48.43 ± 9.12	35.16	0.552
IMT, mm	0.92 ± 0.29	30.71	1.04 ± 0.29	38.87	0.091
hsCRP, mg/L	8.06 ± 6.3	31.48	8.51 ± 5.02	35.70	0.385
INR	1.7 ± 0.49	16.72	1.91 ± 0.5	20.74	0.263

^T^—Mann-Whitney U Test. *—significant difference. BMI, body mass index; NIHSS, National Institutes of Health Stroke Scale; mRS, modified Rankin Scale; CHA_2_DS_2_-VASC, acronym for Congestive Heart Failure, Hypertension, Age (>75 years), Diabetes Mellitus, Stroke/TIA (transient ischemic attack)/TE (thromboembolism), Vascular disease, Age (65–74 years), Sex category; HTN, hypertension; HASBLED, acronym for Hypertension, Abnormal liver/renal function, Stroke, Bleeding, Labile INR, Elderly age (>65 years), Drug/Alcohol usage history/Medication usage with bleeding predisposition; HF NYHA, heart failure New York Heart Association functional classification; DBP, diastolic blood pressure; SBP, systolic blood pressure; TC, total cholesterol; LDLc, low-density lipoprotein cholesterol; HDL-c, high-density lipoprotein cholesterol; HbA1c, glycosylated hemoglobin; TG, triglycerides LAV, left atrial volume; LVEDV, left ventricular end-diastolic volume; LVEF, left ventricular ejection fraction; IMT, intima media thickness; hsCRP, high sensitive C-reactive protein; INR, International Normalized Ratio. Values were expressed as mean ± standard deviation (SD).

**Table 4:** Comparisons of proportions of cardiovascular comorbidities and stroke localization for *MTHFR A1298C* mutation (*n* = 67) using the Chi^2^ Test.

Variable	Without *MTHFR A1298C* Mutation (*n* = 40)	With *MTHFR A1298C* Mutation (*n* = 27)	*p* ^T^
	*n* (%)	*n* (%)	
Sex (male)	14 (35.0%)	13 (48.1%)	0.318
Carotid atheromatosis	23 (57.5%)	16 (59.3%)	0.886
CAD	34 (85.0%)	25 (92.6%)	0.459
PAD	11 (27.5%)	2 (7.4%)	0.059
T2DM	15 (37.5%)	12 (44.4%)	0.618
Ischemic CES:			0.161
Sylvian territor	35 (87.5%)	20 (74.1%)	
Vertebrobasilar territory	4 (10%)	3 (11.1%)	
Hemorrhagic conversion of CES:			
Hemorrhagic infarction	1 (2.5%)	3 (11.1%)	0.295
Parenchymal hematoma	0 (0%)	1 (3.7%)	0.403
Lacunar stroke	9 (22.5%)	14 (51.9%)	0.019 ^*^
Recurrent stroke	12 (30%)	16 (59.3%)	0.024 ^*^

^T^—Chi^2^ Test. *—significant difference. CAD, coronary artery disease; PAD, peripheral vascular disease; T2DM, type II diabetes mellitus; CES, cardioembolic stroke.

**Table 5:** Logistic Regression (using Forward Stepwise (Wald) method) considering *MTHFR C677T* mutation as a dependent variable.

Variables in the Equation								
	B	S.E.	Wald	df	Sig.	Exp(B)	95% C.I. for EXP(B)	
							Lower	Upper
BMI	0.221	0.113	3.836	1	0.050	1.802	1.643	2.950
DBP	0.127	0.046	7.607	1	0.006 ^*^	1.881	1.804	4.964
HbA1c	0.937	0.364	6.629	1	0.010 ^*^	1.392	1.192	7.799
TGL	0.018	0.006	8.565	1	0.003 ^*^	1.982	1.171	3.994
Constant	24.067	7.119	11.429	1	0.001	2.833E10		

*—significant difference.

**Table 6:** Logistic Regression (by Forward Stepwise (Wald) method) using *MTHFR A1298C* as a dependent variable.

Variables in the Equation								
	B	S.E.	Wald	df	Sig.	Exp(B)	95% C.I. for EXP(B)	
							Lower	Upper
TGL	0.017	0.006	9.553	1	0.002 ^*^	2.983	1.972	7.994
Constant	8.430	4.261	3.914	1	0.048	4583.188		

*—significant difference.

**Table 7:** INR Descriptive Statistics.

	*n*	Minimum	Maximum	Mean	Std. Error	Std. Deviation
INR	37	1.04	2.87	1.82	0.082	0.499
